# The Correlation of Total Percent Fat With Alterations in Cholesterol and Triglycerides in Adults

**DOI:** 10.3389/fnut.2022.881729

**Published:** 2022-05-31

**Authors:** Juan Sun, Zimu Zhang, Zhen Liu, Jie Li, Weiming Kang

**Affiliations:** Division of General Surgery, Department of Surgery, Peking Union Medical College Hospital, Chinese Academy of Medical Sciences and Peking Union Medical College, Beijing, China

**Keywords:** NHANES, total percent fat, lipid biomarkers, cholesterol, triglyceride

## Abstract

**Background:**

To evaluate the detailed relationship between total percent fat (TPF) and cardiovascular disease (CVD)-related lipid biomarkers among adults and find a non-invasive indicator for screening and monitoring of the high CVD risk population.

**Methods:**

Data of 13,160 adults were obtained from the National Health and Examination Survey (NHANES) from 1999 to 2018. TPF was assessed by dual-energy x-ray absorptiometry (DXA), and CVD-related lipid biomarkers included total cholesterol (TC), triglyceride (TG), low-density lipoprotein cholesterol (LDL-C), and high-density lipoprotein cholesterol (HDL-C). Multivariable linear regression models were used to examine associations between TPF with four kinds of lipid biomarkers, and smooth curve fittings and generalized additive models were used to address the non-linear relationship between them. The inflection points were calculated by the recursive algorithm when non-linearities were detected and then weighted two-piecewise linear regression models were constructed.

**Results:**

In multivariable regression, increasing TPF was positively associated with TC, TG, and LDL-C and negatively with HDL-C (all *p* < 0.001). In addition, the non-linear relationships between them were also identified by generalized additive models and smooth curve fittings. When further stratified TPF by sex, the fitted smooth curves were nearly inverted U-shaped and U-shaped curves, the inflection points were calculated, and the weighted two-piecewise linear regression models were constructed, respectively. The same results existed between android percent fat and these four lipid biomarkers.

**Conclusions:**

Total percent fat was significantly associated with CVD-related lipid biomarkers in adults, positively with TC, TG, and LDL-C and negatively with HDL-C. It could be used as a non-invasive screener and monitor of high CVD risk population when their TPF values were less than the inflection points.

## Introduction

Cardiovascular disease (CVD) is a growing global health problem. The American Heart Association (AHA) reported in 2021 that 126.9 million (49.2%) adults in the U.S. currently had CVD and that CVD risk increased with age in both men and women (1), which produces immense health and economic burdens in the United States and globally. Clinical lipid biomarkers, such as total cholesterol (TC), triglyceride (TG), low-density lipoprotein cholesterol (LDL-C), and high-density lipoprotein cholesterol (HDL-C), are essential for the evaluation of the risk of developing CVD. For example, TG concentration had a prognostic value for assessing coronary heart disease (CHD) risk, especially when used in combination with HDL-C and LDL-C (2), increased TC and LDL-C may also be risk factors for CVD (3), HDL-C was reported to be a strong, consistent, and independent predictor of CVD (4). Besides, overexpression of apolipoprotein A-I (ApoA-I; a major HDL protein) was proved to be atheroprotective, a study by Rached et al. showed that individuals with familial ApoA-I deficiency have diminished atheroprotective activities of HDL-C due to altered ApoA-I function (5), a study by Sontag indicated that ApoA-I was atheroprotective only in certain genetic contexts in mice (6), and a review by Lee-Rueckert also underlined the difficulty of translating HDL-C functioning from mouse to human data (7).

Obesity is characterized by the increase in the volume or number of fat cells in the body, resulting in an abnormal increase in the total fat and excessive fat deposition in some parts. Traditionally, obesity is estimated by body mass index (BMI), however, total percent fat (TPF) as measured by dual-energy x-ray absorptiometry (DXA) is a more accurate method to measure obesity and is more correctly correlated with CVD risks (8, 9), such as hypertension, diabetes, and dyslipidemia (10, 11). However, the relationship between TPF and CVD risks is not consistent in different populations (12, 13), such as different TPF in different sex, which suggests that sex should be considered when conducting relevant studies on TPF (14).

However, few studies have demonstrated the relationship of TPF with alterations in cholesterol and TGs. Are they positively or negatively correlated and can TPF be used as a non-invasive monitoring indicator of CVD in the human microenvironment? In addition, what is the effect of sex on their relationship? Since women naturally have a higher fat percentage, which does not directly correlate to higher risk, as they also have atheroprotective estrogen-mediated effects. In this study, we evaluated the relationship of TPF with four kinds of traditional lipid biomarkers based on a large and representative U.S. general population from the National Health and Nutrition Examination Survey (NHANES) from 1999 to 2018. We also stratified TPF by sex to expound on the different inflection points of lipid biomarkers with the growth trend of TPF.

## Materials and Methods

### Study Population

A cross-sectional analysis of data from 10 cycles of the NHANES from 1999 to 2018 was conducted in this study. NHANES is a continuous surveillance survey conducted by the Centers for Disease Control and Prevention (CDC) and the National Center for Health Statistics (NCHS) to assess the health and nutritional status of the U.S. population. It uses a stratified, multistage probability sampling design to obtain nationally representative estimates and includes a household interview followed by additional assessments at mobile examination centers (MECs).

A total of 59,204 participants who were aged ≥18 years old were enrolled from the NHANES 1999–2018 database. After the exclusion of 28,628 participants without TPF data, 1,691 participants without TC data, 15,050 without TG data, and 675 without LDL-C data, 13,160 participants remained in the final analysis.

### Variables

The exposure variable of this study was TPF, measured by DXA of the whole body. The outcome variables were TC, TG, LDL-C, and HDL-C, which were all gained from the NHANES laboratory part. A detailed description of the laboratory protocols can be found on the NHANES website (http://www.cdc.gov/nchs/nhanes/).

The following categorical variables were included in our analysis as covariates: sex, race, education level, marital status, vigorous work activity, hypertension (ever told by a doctor that you have high blood pressure), diabetes (ever told by a doctor that you have diabetes), smoking status (whether smoked at least 100 cigarettes in life), arthritis status (doctor ever told you had arthritis), CHD status (has a doctor or other health professional ever told you that you had CHD), and liver disease status [ever told you had any liver condition, such as viral hepatitis (hepatitis A, B, and C); autoimmune liver disease (primary biliary cirrhosis, autoimmune hepatitis, and sclerosing cholangitis); genetic liver diseases (alpha-1-antitrypsin deficiency, hemochromatosis, and Wilson's disease); drug-or-medication-induced liver disease; alcoholic liver disease; non-alcoholic fatty liver disease; fatty liver disease; liver cancer; liver cyst; liver abscess; liver fibrosis; and liver cirrhosis]. The continuous covariates were age, BMI, the ratio of family income to poverty, and android percent fat (the android area was defined as the lower trunk area bounded by two lines: the pelvic horizontal cut line on its lower side, and a line automatically placed above the pelvic line). Detailed information on TPF, TC, TG, LDL-C, HDL-C, and other covariates is also publicly available on the NHANES website.

### Statistical Analysis

The NHANES sample weights were used as recommended by the NCHS. All analyses were performed with package R (http://www.Rproject.org) and Empower Stats (http://www.empowerstats.com), with a value of *p* < 0.05 considered statistically significant. Multivariable linear regression models were performed to evaluate the associations between TPF and TC, TG, LDL-C, and HDL-C. Three models were built, which are as follows: unadjusted model 1, minimally adjusted model 2 (adjusted for age, sex, and race), and fully adjusted model 3 (adjusted for age, sex, race, BMI, hypertension, diabetes, smoking status, and vigorous work activity). The smooth curve fittings and generalized additive models were used to address the non-linear relationship between them. When non-linearity was detected, we further calculated the inflection point using a recursive algorithm and constructed a weighted two-piecewise linear regression model.

## Results

A total of 13,160 participants aged ≥18 years old were included in this study. The weighted distributions of the characteristics according to sex are shown in Table 1. Compared with males, females had higher BMI, TC, HDL-C, TPF, and android percent fat, higher percentages of high education level and ever had arthritis but lower ratios of family income to poverty, TG, and LDL-C, and lower percentages of vigorous work activity, smoking status, CHD status, and liver disease status.

**Table 1:** The characteristics of participants.

	**Males (6,613)**	**Females (6,547)**
Age (years)	41.02 ± 14.74	42.48 ± 15.07
BMI (kg/m^2^)	28.03 ± 5.79	28.42 ± 7.20
Ratio of family income to poverty	3.05 ± 1.62	2.92 ± 1.66
Total cholesterol (mmol/L)	4.96 ± 1.02	5.06 ± 1.03
Triglyceride (mmol/L)	1.46 ± 0.82	1.26 ± 0.71
LDL-C (mmol/L)	3.04 ± 0.91	2.98 ± 0.90
HDL-C (mmol/L)	1.25 ± 0.33	1.50 ± 0.41
Android fat percent (%)	31.21 ± 8.64	38.19 ± 8.91
Total percent fat (%)	27.44 ± 6.27	39.19 ± 6.68
**Race (%)**
Hispanic	15.48	13.96
Non-hispanic white	67.60	67.30
Non-hispanic black	9.95	11.86
Others	6.97	6.88
**Hypertension (%)**
No	75.62	75.45
Yes	24.38	24.55
**Diabetes (%)**
No	93.18	93.21
Yes	6.82	6.79
**Vigorous work activity (%)**
No	61.17	73.74
Yes	38.83	26.26
**Smoking status (%)**
No	46.90	60.32
Yes	53.10	39.68
**Education level (%)**
High school and below	44.59	39.65
More than high school	55.41	60.35
**Marital status (%)**
Married or living with a partner	65.17	62.23
Others	34.83	37.77
**Arthritis status (%)**
No	83.91	77.40
Yes	16.09	22.60
**Coronary heart disease status (%)**
No	96.96	98.68
Yes	3.04	1.32
**Liver disease status (%)**
No	95.97	97.09
Yes	4.03	2.91

*Mean ± SD for continuous variables. % for categorical variables*.

### Associations Between TPF and Lipid Biomarkers

Even with the adjustment for age, sex, race, BMI, hypertension, diabetes, smoking status, and vigorous work activity, we found significant correlations between TPF and lipid biomarkers (TC, TG, LDL-C, and HDL-C). The results are shown in Table 2. Similar results were found in the association of android percent fat with these lipid biomarkers, and the results are shown in the Supplementary Material.

**Table 2:** Associations between total percent fat (%) and lipid biomarkers (mmol/L).

	**Exposure: total percent fat (%)**		
	**Model 1 β (95% CI) *P-*value**	**Model 2 β (95% CI) *P-*value**	**Model 3 β (95% CI) *P*-value**
**Outcomes (mmol/L)**
Total cholesterol	0.02 (0.02, 0.02) <0.0001	0.01 (0.01, 0.02) <0.0001	0.02 (0.02, 0.03) <0.0001
Triglyceride	0.01 (0.01, 0.01) <0.0001	0.03 (0.03, 0.03) <0.0001	0.01 (0.01, 0.02) <0.0001
LDL-C	0.01 (0.01, 0.01) <0.0001	0.02 (0.02, 0.02) <0.0001	0.02 (0.02, 0.03) <0.0001
HDL-C	0.00 (0.00, 0.00) 0.0018	−0.02 (-0.02,−0.02) <0.0001	−0.01 (-0.01,−0.01) <0.0001

*Model 1, no covariate was adjusted; Model 2, age, sex, and race were adjusted; Model 3, age, sex, race, BMI, hypertension, diabetes, smoking status, and vigorous work activity were adjusted*.

### The Inflection Points of the Non-linear Relationship Between TPF and Lipid Biomarkers

**Figure 1:**
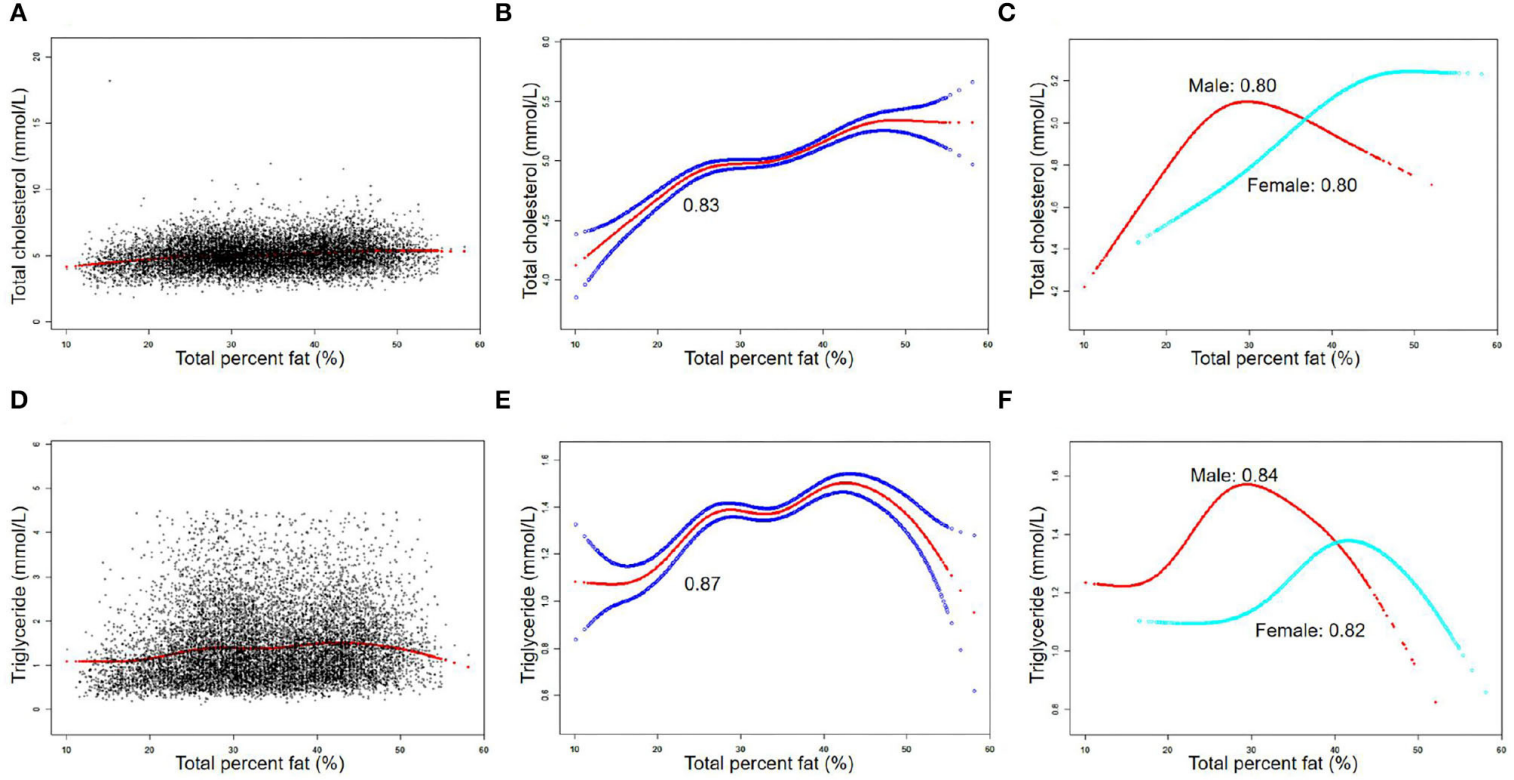
The association between total percent fat (%) and total cholesterol, triglyceride (mmol/L). **(A,D)** Each black point represents a sample. **(B,E)** Solid red line represents the smooth curve fit between variables. Blue bands represent the 95% of confidence interval (CI) from the fit. **(C,F)** Stratified by sex. The correlation coefficients of **(B,C,E,F)** were described in the corresponding figures. Age, sex, race, BMI, hypertension, diabetes, smoking status, and vigorous work activity were adjusted [**(C)** and **(F)** were not adjusted by sex].

**Figure 2:**
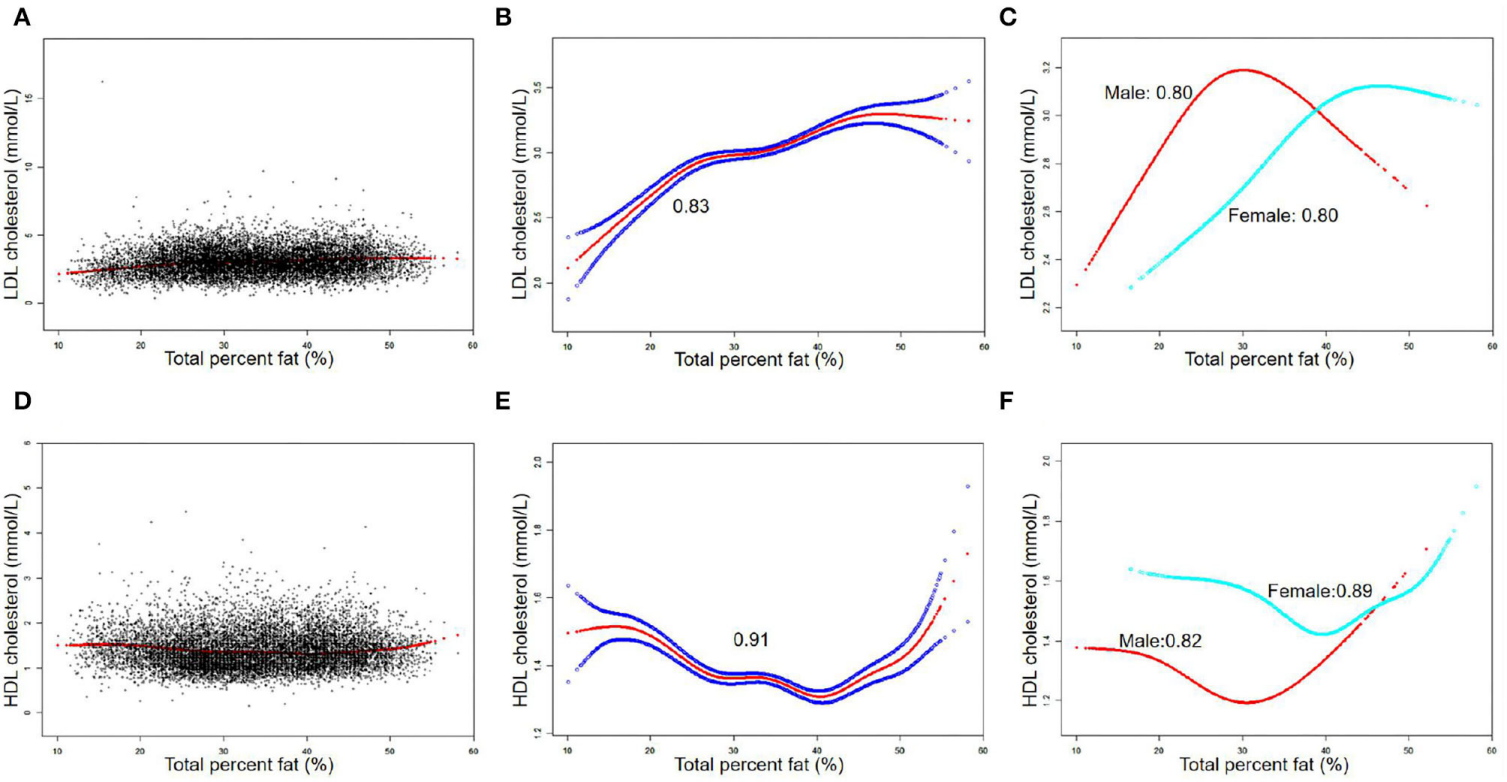
The association between total percent fat (%) and low-density lipoprotein cholesterol (LDL-C), high-density lipoprotein cholesterol (HDL-C) (mmol/L). **(A,D)** Each black point represents a sample. **(B,E)** Solid red line represents the smooth curve fit between variables. Blue bands represent the 95% of confidence interval (CI) from the fit. **(C,F)** Stratified by sex. The correlation coefficients of **(B,C,E,F)** were described in the corresponding figures. Age, sex, race, BMI, hypertension, diabetes, smoking status, and vigorous work activity were adjusted [**(C)** and **(F)** were not adjusted by sex].

The associations between TPF and lipid biomarkers (TC, TG, LDL-C, and HDL-C) were further confirmed by weighted generalized additive models and smooth curve fittings (Figures 1, 2). Non-linear relationships of TPF with lipid biomarkers were detected. Besides, when stratified by sex, we found that the associations were inverted U-shaped and U-shaped curves and further calculated their inflection points. Men: TC: 27.2%, TG: 28.1%, LDL-C: 28.0%, and HDL-C: 28.8%; women: TC: 46.1%, TG: 43.7%, LDL-C: 39.5%, and HDL-C: 39.5% (Table 3). Similar non-linear relationships of android percent fat with lipid biomarkers are shown in the Supplementary Material.

**Table 3:** Threshold effect analysis of total percent fat (%) and lipid biomarkers (mmol/L) using the two-piecewise linear regression model.

	**Exposure: Total percent fat (%)**			
		** < Inflection point**	**> Inflection point**	**Log**
**Outcomes (mmol/L)**	**Inflection point**	**Adjusted β (95% CI) *P*-value**	**Adjusted β (95% CI) *P*-value**	**likelihood ratio**
**Males**
Total cholesterol	27.2	0.06 (0.05, 0.06) <0.0001	−0.02 (−0.03, −0.01) 0.0002	<0.001
Triglyceride	28.1	0.04 (0.03, 0.04) <0.0001	−0.03 (−0.03, −0.02) <0.0001	<0.001
LDL-C	28.0	0.05 (0.05, 0.06) <0.0001	−0.02 (−0.03, −0.01) <0.0001	<0.001
HDL-C	28.8	−0.02 (−0.02, −0.02) <0.0001	0.01 (0.01, 0.01) <0.0001	<0.001
**Females**
Total cholesterol	46.1	0.03 (0.02, 0.04) <0.0001	−0.02 (−0.04, 0.01) 0.1832	<0.001
Triglyceride	43.7	0.02 (0.02, 0.03) <0.0001	−0.02 (−0.03, −0.01) <0.0001	<0.001
LDL-C	39.5	0.04 (0.03, 0.05) <0.0001	0.00 (−0.01, 0.01) 0.8696	<0.001
HDL-C	39.5	−0.02 (−0.02, −0.01) <0.0001	0.02 (0.01, 0.02) <0.000	<0.001

*Age, sex, race, BMI, hypertension, diabetes, smoking status, and vigorous work activity were adjusted. (Sex was not adjusted when stratified by sex)*.

Detailed speaking, in men, for a TPF <27.2%, every 1% increase in TPF was associated with 0.06 mmol/L higher TC (95% CI: 0.05, 0.06), but 0.02 mmol/L lower TC when TPF >27.2% (95% CI: −0.03, −0.01). In addition, for a TPF <28.1%, every 1% increase in TPF was associated with 0.04 mmol/L higher TG (95% CI: 0.03, 0.04), but 0.03 mmol/L lower TG when TPF >28.1% (95% CI: −0.03, −0.02). Moreover, for a TPF <28.0%, every 1% increase in TPF was associated with 0.05 mmol/L higher LDL-C (95% CI: 0.05, 0.06), but 0.02 mmol/L lower LDL-C when TPF >28.0% (95% CI: −0.03, −0.01). In HDL-C, it was a U-shaped curve, for a TPF <28.8% in men, every 1% increase in TPF was associated with 0.02 mmol/L lower HDL-C (95% CI: −0.02, −0.02), but 0.01 mmol/L higher when TPF >28.8% (95% CI: −0.03, −0.01).

In women, for a TPF <46.1%, every 1% increase in TPF was associated with 0.03 mmol/L higher TC (95% CI: 0.02, 0.04), but it was not statistically significant when TPF >46.1% (95% CI: −0.04, 0.01). In addition, for a TPF <43.7%, every 1% increase in TPF was associated with 0.02 mmol/L higher TG (95% CI: 0.02, 0.03), but 0.02 mmol/L lower TG when TPF >43.7% (95% CI: −0.03, −0.01). In terms of LDL-C and HDL-C, for a TPF <39.5%, every 1% increase in TPF was associated with 0.04 mmol/L higher LDL-C (95% CI: 0.03, 0.05) and 0.02 mmol/L lower HDL-C (95% CI: −0.02, −0.01), but no statistically significant in LDL-C (95% CI: −0.01, 0.01) and 0.02 mmol/L higher in HDL-C was found (95% CI: 0.01, 0.02) when TPF >39.5%.

## Discussion

The results of this study showed that TPF was significantly associated with lipid biomarker levels in a nationally representative sample of U.S. adults. In detail, TPF was positively associated with TC, TG, and LDL-C and negatively associated with HDL-C. Moreover, the non-linear relationships between them stratified by sex were also identified by generalized additive models and smooth curve fittings, with their different inflection points calculated: men: TC: 27.2%, TG: 28.1%, LDL-C: 28%, and HDL-C: 28.8%; women: TC: 46.1%; TG: 43.7%; LDL-C: 39.5%; and HDL-C: 39.5%.

Being overweight or obese as an adult increases the risk of CVD by 2- to 3-fold in women and 1- to 2-fold in men (15). TC, TG, LDL-C, and HDL-C are clinically recognized as lipid biomarkers for predicting and monitoring CVD, but invasively blood drawing is needed because they are all tested from serum. However, TPF can directly and non-invasively reflect the body fat composition to evaluate obesity and has proved to be associated with CVD (10–12, 16). In many research and screening settings, TPF measured by DXA may be useful to screen, monitor, and manage of high-risk population of CVD if it could be shown to be valid estimators of lipid biomarkers and if the associations were sufficient to identify those at the highest risks of increased or decreased serum lipid biomarkers. From a clinical standpoint, the most typical lipid profile seen in obese individuals is the increase of fasting plasma TG, the decrease of HDL-C, and the marginal increase of LDL-C in adults (17). In this study, we found positive associations between TPF and TC, TG, and LDL-C and a negative association with HDL-C in adults too. Furthermore, because of the different TPF in men and women, we matched the non-linear relationships in the subgroup of sex and found different inflection points. From our results, we also draw a conclusion that TPF could be used to monitor the changes of lipid biomarkers in serum and further evaluate the risk of CVD when the TPF was approximately <28% in men and <40% in women (the detailed TPF values for four lipid biomarkers were as follows: men: TC: 27.2%, TG: 28.1%, LDL-C: 28%, and HDL-C: 28.8%; women: TC: 46.1%; TG: 43.7%; LDL-C: 39.5%; and HDL-C: 39.5%.). However, when TPF was higher than the inflection points, such monitoring cannot be done anymore because lipid biomarkers turned to a low CVD risk direction (or no statistically significant in TC and LDL-C of women) as TPF was increased.

Similar to our findings, Haarbo et al. studied postmenopausal women and found positive associations between central fat and cholesterol, TGs, and LDL-C and a negative association with HDL-C (18, 19). Moreover, then Daniels et al. found that a greater android fat distribution was significantly and independently related to plasma TG and HDL-C in a cross-sectional study of 127 children and adolescents 9–17 years of age (20). Later in 2012, a study by Addo et al. showed that adiposity is measured by both skinfold thicknesses, and DXA whole-body fat weight is positively and significantly associated with serum TG levels in a nationally representative sample of 1,505 U.S. adolescents (21). In the study of Hetherington-Raut in 2018, 239 Hispanic girls aged 9–12 years old were included and indicated that partial correlations for the percentage of total fat in the gynoid and leg regions with insulin, Homeostatic Model Assessment for Insulin Resistance (HOMA-IR), TG, and LDL-C were negative and positive with HDL-C (22). However, these findings were limited by the small sample size and a focus on specific individuals, and some have only evaluated the specific areas of the body fat, some just simply expounded on the general correlation between TPF and lipid biomarkers but not the detailed inflection point.

The mechanism by which TPF influences these CVD-related lipid biomarkers is not completely understood. However, studies have verified that as total body fat mass increases, the rate of fat increase varied in different regions, with more fat deposited centrally and less periphery (23), and some metabolic alterations were related to more central fat deposition in adults (20). Individuals with excess central fat cause insulin resistance, promoting visceral adipocytes to release excessive free fatty acids absorbed by the liver as raw materials for the synthesis of TG (24). Increased insulin resistance further leads to reduced lipoprotein lipase and increased hepatic lipase, resulting in the decreased maturation and increased catabolism of HDL-C, respectively (25). Other proposed mechanisms include the dysregulation of adipokines or inflammatory cytokines, accelerating the abnormal metabolism of the endothelium (26), and the adverse effect of insulin on sympathetic nerve activity (27).

## Conclusion

This study indicated that TPF was significantly associated with CVD-related lipid biomarkers in adults, positively with TC, TG, and LDL-C and negatively with HDL-C. Furthermore, the non-linear relationships between them were fitted with the smooth curve when stratified by sex and all had their inflection points, which merits further research to elucidate the burden of obesity and CVD health.

## Strengths and Limitations

The main strengths of this study are the availability of a large, nationally representative population of U.S. adults with complete data on TPF and lipid biomarkers from NHANES. Additionally, the availability of body composition measures offers additional information above traditional measures of adiposity. A standardized protocol was also used to multiply imputed data sets for participants with missing body composition data, resulting in a more complete analytic dataset. More importantly, a large enough sample size was used allowing us to make the subgroup by sex and show the distinct but neglected pattern of TPF and lipid biomarkers, which was never reported in the previous studies.

This study also has several limitations. Firstly, it is the cross-sectional design of our study, which limits the inference of a causal correlation between TPF and lipid biomarkers among adults. However, the observed associations are still valid at a single time point and should represent the concurrent associations fairly, which are often the focus of many nutritional assessment or surveillance programs. However, further basic mechanism research and large sample prospective study are still needed to identify the exact mechanism between them. Secondly, some NHANES participants were not eligible for a DXA scan because of excessive weight or height or other reasons, so the estimates in this study might not fully represent the TPF among the general population. Thirdly, there remains the possibility of bias caused by other potential confounding factors that we did not adjust for, for instance, women and men differ naturally in their TPF and women have atheroprotective effects of Estrogen.

## Data Availability Statement

Publicly available datasets were analyzed in this study. These data can be found here: http://www.cdc.gov/nchs/nhanes/.

## Author Contributions

JS, ZZ, ZL, and JL contributed to the data collection and analysis. JS contributed in writing of the manuscript. WK contributed to the study design and polish and review of the manuscript. All authors contributed to the article and approved the submitted version.

## Funding

CSCO-ROCHE Research Fund No. Y-2019 Roche-015; Beijing Xisike Clinical Oncology Research Foundation Y-HS2019-43; Wu Jieping Medical Foundation No. 320. 6750.19020; 2017 Beijing Municipal Science and Technology Project: D171100006517004; and CAMS Innovation Fund for Medical Sciences: No. 2020-I2M-C&T-B-027.

## Conflict of Interest

The authors declare that the research was conducted in the absence of any commercial or financial relationships that could be construed as a potential conflict of interest.

## Publisher's Note

All claims expressed in this article are solely those of the authors and do not necessarily represent those of their affiliated organizations, or those of the publisher, the editors and the reviewers. Any product that may be evaluated in this article, or claim that may be made by its manufacturer, is not guaranteed or endorsed by the publisher.
