# Supplementary material for: The Correlation of Total Percent Fat With Alterations in Cholesterol and Triglycerides in Adults
Source: Front Nutr. 2022 May 31;9:881729. doi: 10.3389/fnut.2022.881729 (PMC9197496; doi:10.3389/fnut.2022.881729)
Supplement: Supplementary file 1 [file Data_Sheet_1.PDF]

Supplementary Table 1

Associations between android percent fat (%) and lipid biomarkers (mmol/L)

	Exposure: android percent fat (%)		
	Model 1	Model 2	Model 3
	β (95% CI) P value	β (95% CI) P value	β (95% CI) P value
Outcomes (mmol/L)			
Total cholesterol	0.02 (0.02, 0.02)	0.01 (0.01, 0.01)	0.02 (0.02, 0.03)
	<0.0001	<0.0001	<0.0001
Triglyceride	0.02 (0.02, 0.02)	0.02 (0.02, 0.03)	0.02 (0.02, 0.02)
	<0.0001	<0.0001	<0.0001
LDL cholesterol	0.02 (0.02, 0.02)	0.02 (0.01, 0.02)	0.02 (0.02, 0.02)
	<0.0001	<0.0001	<0.0001
HDL cholesterol	-0.01 (-0.01, -0.01)	-0.02 (-0.02, -0.02)	-0.01 (-0.01, -0.01)
	<0.0001	<0.0001	<0.0001

Notes: Model 1: no covariate was adjusted. Model 2: age, sex, race were adjusted. Model 3: age, sex, race, BMI, hypertension, diabetes, smoking status, vigorous work activity were adjusted.

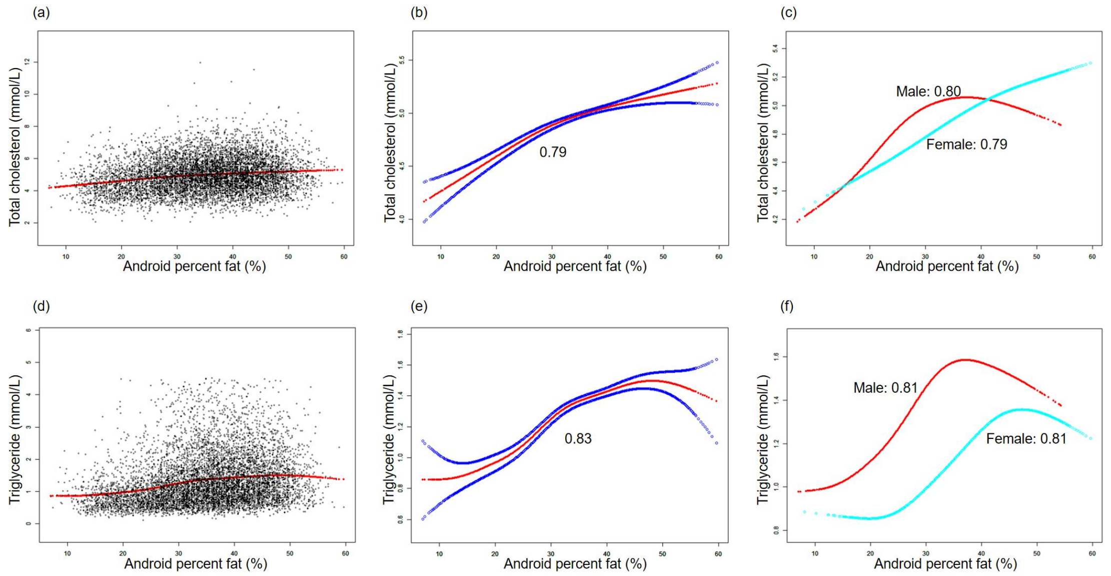

Supplementary Figure 1 The association between android percent fat (%) and total cholesterol, triglyceride (mmol/L). **(a) (d)** Each black point represents a sample. **(b) (e)** Solid rad line represents the smooth curve fit between variables. Blue bands represent the 95% of confidence interval from the fit. **(c) (f)** Stratified by sex. The correlation coefficients of (b), (c), (e), (f) were described in the corresponding figures.

Age, sex, race, BMI, hypertension, diabetes, smoking status, vigorous work activity were adjusted (c and f were not adjusted by sex).

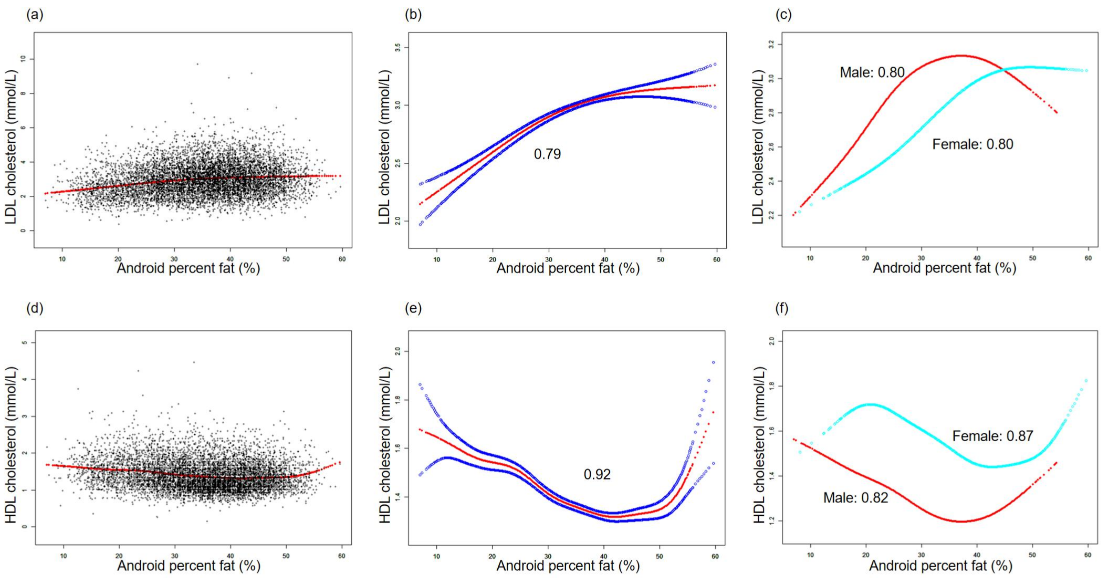

Supplementary Figure 2 The association between android percent fat (%) and LDL cholesterol, HDL cholesterol (mmol/L). **(a) (d)** Each black point represents a sample. **(b) (e)** Solid rad line represents the smooth curve fit between variables. Blue bands represent the 95% of confidence interval from the fit. **(c) (f)** Stratified by sex. The correlation coefficients of (b), (c), (e), (f) were described in the corresponding figures.

Age, sex, race, BMI, hypertension, diabetes, smoking status, vigorous work activity were adjusted (c and f were not adjusted by sex).

Supplementary Table 2

Table 3 Threshold effect analysis of android percent fat (%) and lipid biomarkers (mmol/L) using the two piecewise linear regression model

		Exposure: Android percent fat (%)		
	Inflection point	< Inflection point<br>Adjusted β (95% CI) P value	> Inflection point<br>Adjusted β (95% CI) P value	Log likelihood ratio
Outcomes (mmol/L)				
**Males**				
Total cholesterol	30.1	0.04 (0.04, 0.05)<br><0.0001	-0.00 (-0.01, 0.00)<br>0.2913	<0.001
Triglyceride	35.6	0.03 (0.02, 0.03)<br><0.0001	-0.02 (-0.03, -0.01)<br>0.0022	<0.001
LDL cholesterol	30.1	0.04 (0.04, 0.05)<br><0.0001	-0.00 (-0.01, 0.00)<br>0.2187	<0.001
HDL cholesterol	35.7	-0.01 (-0.02, -0.01)<br><0.0001	0.01 (0.00, 0.01)<br>0.0002	<0.001
**Females**				
Total cholesterol	45.3	0.03 (0.02, 0.04)<br><0.0001	-0.01 (-0.03, 0.01)<br>0.3981	<0.001
Triglyceride	45.4	0.03 (0.02, 0.03)<br><0.0001	-0.01 (-0.03, -0.00)<br>0.0235	<0.001
LDL cholesterol	45.1	0.03 (0.02, 0.03)<br><0.0001	-0.02 (-0.04, -0.00)<br>0.0210	<0.001
HDL cholesterol	43.0	-0.01 (-0.02, -0.01)<br><0.0001	0.01 (0.01, 0.02)<br><0.0001	<0.001

Notes: Age, sex, race, BMI, hypertension, diabetes, smoking status, vigorous work activity were adjusted. (Sex was not adjusted when stratified by sex).
